# C-X-C Motif Ligand 1 (CXCL1) from melanoma cells down-regulates the invasion of their metastatic melanoma cells

**DOI:** 10.18632/oncotarget.25783

**Published:** 2018-07-24

**Authors:** Takaharu Hatano, Masakazu Yashiro, Heishiro Fujikawa, Hisashi Motomura

**Affiliations:** ^1^ Department of Plastic and Reconstructive Surgery, Osaka City University Graduate School of Medicine, Osaka, Japan; ^2^ Molecular Oncology and Therapeutics, Osaka City University Graduate School of Medicine, Osaka, Japan; ^3^ Cancer Center for Translational Research, Osaka City University Graduate School of Medicine, Osaka, Japan

**Keywords:** cytokine, CXCL1, down-regulate, melanoma, metastasis

## Abstract

The surgical resection of a primary melanoma is sometimes followed by the immediate development of distant metastases, suggesting that the primary melanoma might control the metastatic process. We hypothesized that a paracrine factor(s) from primary melanoma cells might regulate the progression of metastasizing melanoma cells. Here we attempted to identify the factor(s) from primary melanoma cells that regulate the invasion ability of metastatic melanoma cells. We used two mouse melanoma cell lines, B16 and B16/BL6, that latter of which is a subline of B16 melanoma and shows high metastatic potential to lung. We investigated the interaction between the parent B16 cells and daughter B16/BL6 cells by invasion assay, cell morphology, cytokine array, RT-PCR, and gelatin-zymography. The conditioned medium (CM) from B16 significantly (p=0.02) inhibited the invasion ability of B16/BL6 cells. The morphology of the B16/BL6 cells was changed from bipolar shape to a multipolar shape following the addition of the CM from B16. The B16 cells produced high levels of C-X-C motif ligand 1 (CXCL1), CXCL10, and M-CSF compared to the B16/BL6 cells. CXCL1 significantly (p=0.01) decreased the invasion ability of B16/BL6 cells, but CXCL10 and M-CSF did not. The invasion-inhibitory activity of the CM from B16 was significantly (p=0.046) suppressed following the addition of a neutralizing anti-CXCL1 antibody. The CM of B16 and CXCL1 increased the *E-cadherin* mRNA level and decreased MMP2 activity of B16/BL6 cells. These findings suggested that primary melanoma cells might down-regulate the invasion activity of metastatic melanoma cells through CXCL1 signaling.

## INTRODUCTION

The incidence of melanoma is approx. 0.93 per 100,000 in Japan, and the incidence in Australia and the U.S. is 45 per 100,000 and 22 per 100,000, respectively [1]. Melanoma represents approx. 4% of all cancers in the U.S., and it was estimated that 59,580 melanomas were newly diagnosed in 2005 [2]. Although early-stage melanoma can be curatively treated by a surgery alone [3], most melanoma cells spread rapidly and quickly reach the advanced stage with distant metastases. The prognosis of patients with malignant melanoma is thus extremely poor because of the frequent metastases to distant organs such as lung and lymph nodes. Molecular-targeted therapy based on the characterization of the metastasis of melanoma is thus desirable.

There are clinical reports that distant metastases developed immediately after the removal of primary melanomas [4, 5], suggesting that the metastatic process might be controlled by the primary tumor. The potential of a primary melanoma to inhibit the development of distant metastases has also been discussed based on experimental evidence [6–8]. Kirstein *et al.* reported that the presence of B16F10 melanoma cells significantly restricted the numbers and sizes of experimental lung metastases [6]. Kubo *et al.* reported that B16 primary tumors inhibited the development of secondary B16 tumors in the lung and in the peritoneum and suppressed the experimental metastases of E0771 breast cancer cells to lung [7]. Hanniford *et al.* reported that the microRNAs miR-382 and miR-516b in primary melanoma suppress the invasion and metastasis of melanoma cells [8].

An interaction between primary tumor cells and metastatic cells might exist during the multistep cascade of distant metastases, via soluble factors. We hypothesized that one or more paracrine factors from primary melanoma cells might regulate the progression of metastasizing melanoma cells. Here, we investigated the invasion and growth interaction between primary malignant melanoma cells and metastatic melanoma cells. To the best of our knowledge, this is the first study to show that the chemokine (C-X-C motif) ligand 1 (CXCL1) from primary melanoma cells might down-regulate the invasion ability of metastatic melanoma cells.

## RESULTS

### Effect of CM from melanoma cells on the invasion ability of melanoma cells

**Figure 1:**
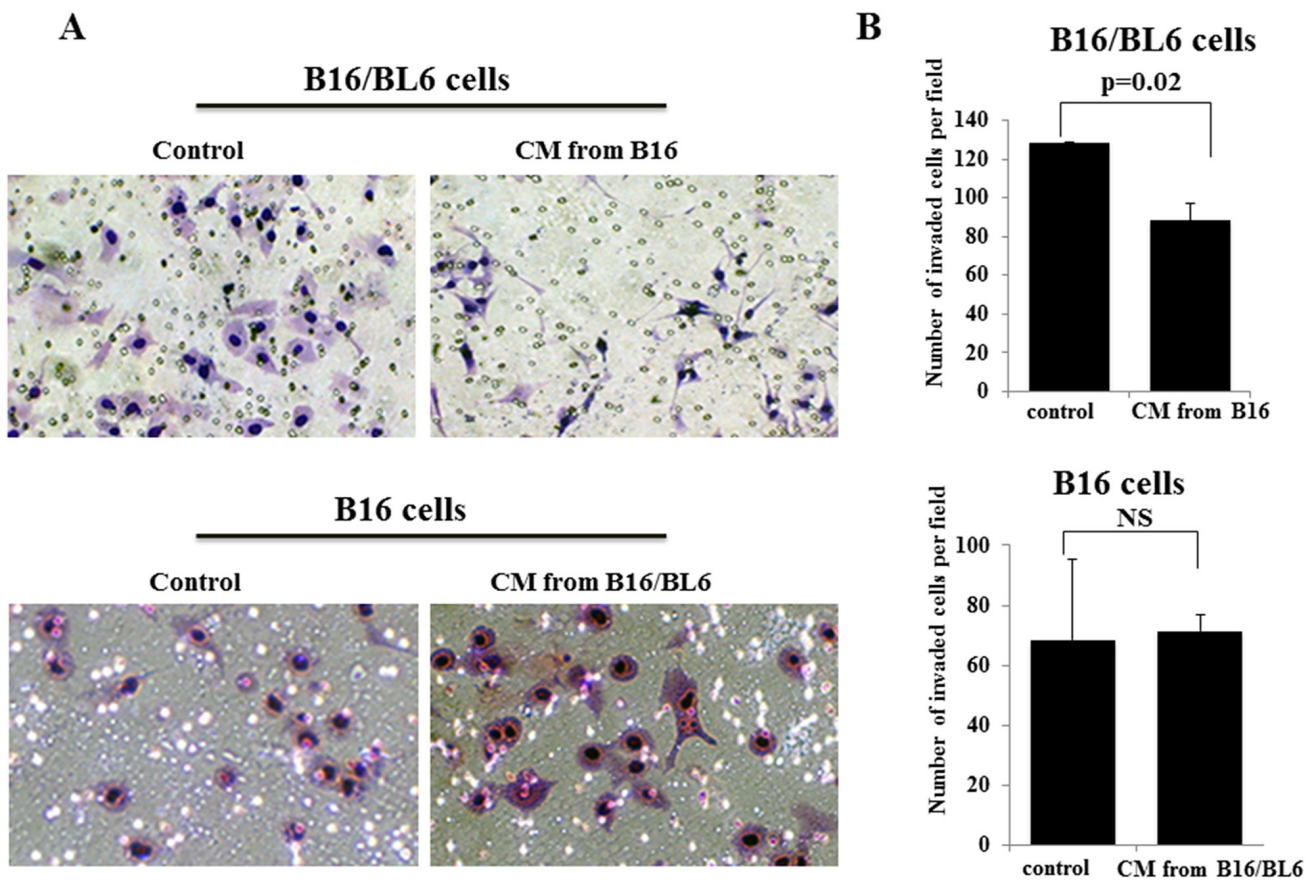
Effects of CM on the invasion of B16/BL6 cells and B16 cells **(A)** Representative picture of invasion assay results. The number of invading B16/BL6 cells was low in the presence of CM from B16, in compared to that of the control. In contrast, the number of invading B16 cells was not different between the control and the CM from B16/BL6. **(B)** CM from B16 significantly down-regulated the invasion ability of B16/BL6 cells (p=0.02), in compared to the control. The invasion ability of B16 cells was not different between the control and the addition of the CM from B16/BL6. The results are the mean of three independent experiments. Bars: SD.

**Figure 2:**
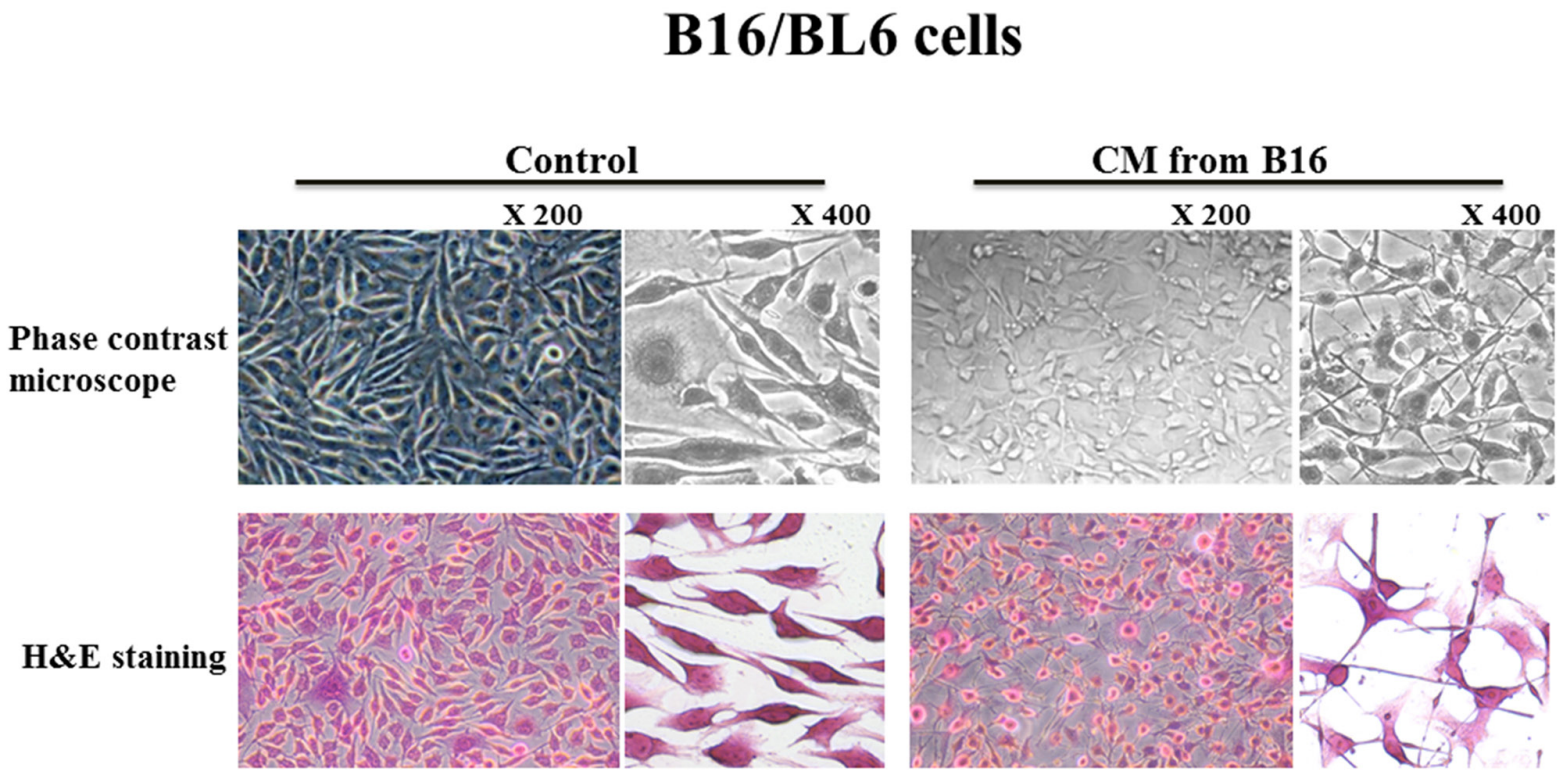
Effect of CM on the morphology of B16/BL6 cells The CM from B16 changed the shape of the B16/BL6 cells from bipolar to multipolar.

The number of invaded B16/BL6 cells following the addition of CM from B16 was lower than that of the control, whereas the number of invaded B16 cells was not affected by the CM from B16/BL6 (Figure 1A). The CM from B16 significantly (p=0.02) decreased the number of invading B16/BL6 cells. In contrast, the CM from B16/BL6 did not affect the invasion ability of B16 cells (Figure 1B). Each CM did not affect the proliferation of B16/BL6 cells or B16 cells (Supplementary Figure 1A). B16/BL6 cells showed a morphologic change from the bipolar shape to the multipolar shape following the addition of the CM from B16 (Figure 2). In contrast, the CM from B16/BL6 did not affect the morphologic features of B16 cells (data not shown).

### Cytokine production from melanoma cells

**Figure 3:**
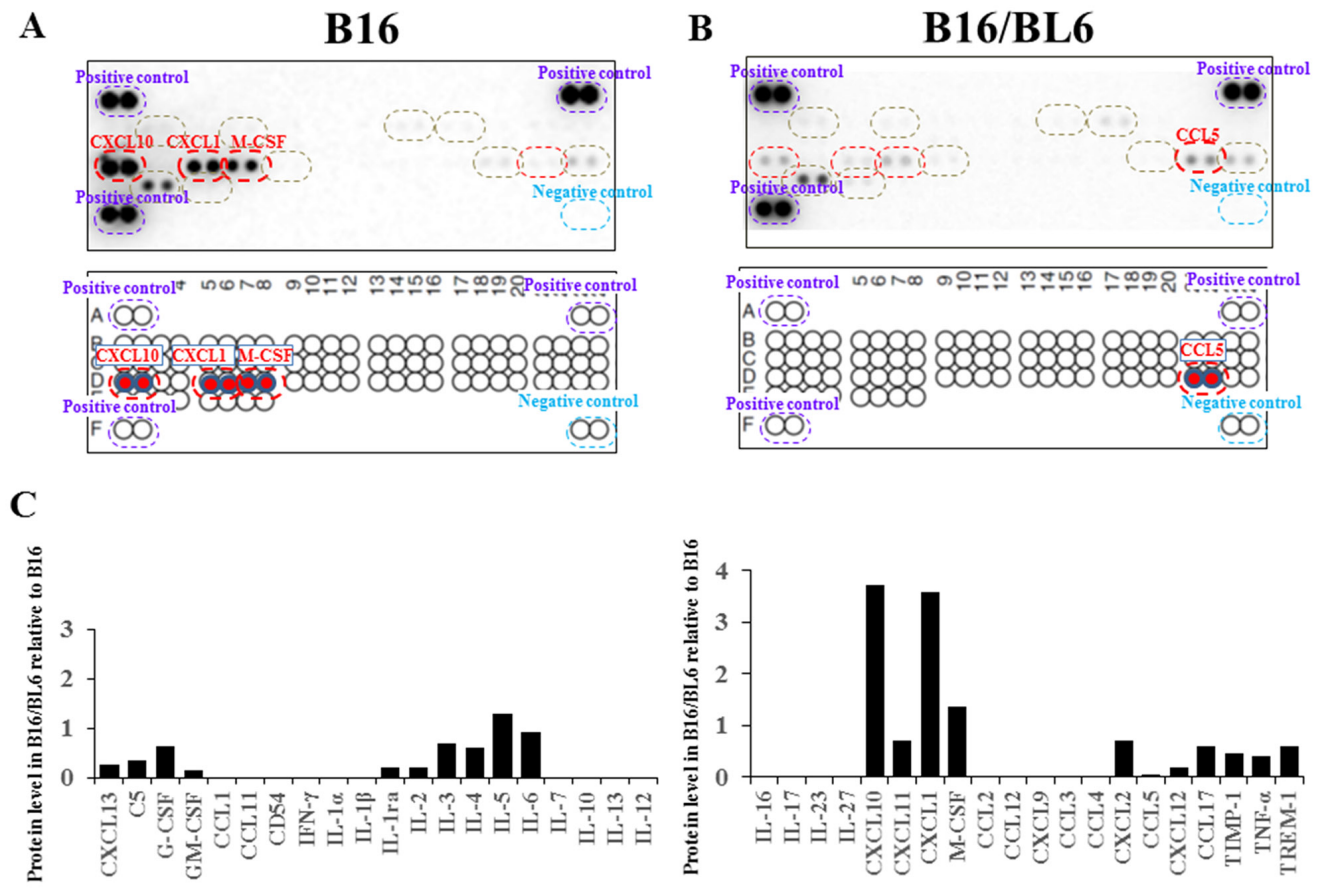
Cytokine analysis of CM **(A, B)** Cytokine array. The levels of CXCL1, CXCL10, and M-CSF were higher in the CM from B16 compared to the CM from B16/BL6 (A). CCL5 was higher in the CM from B16/BL6 compared to the CM from B16 (B). **(C)** Signal intensity of cytokine array.

We used the Mouse Cytokine Array which detects the relative levels of 40 different cytokines. B16 cells produced high levels of CXCL1, CXCL10, and M-CSF compared to the B16/BL6 cells (Figure 3A). In contrast, the B16/BL6 cells produced a high level of CCL5 compared to the B16 cells (Figure 3B). Although C5/C5a, GM-CSF, IFN-γ, IL-1α, CCL2/MCP-1, CXCL2, SDF-1, TIMP-1, and TNF-α, were produced from both cell lines, no difference of production level was found between the B16 cells and B16/BL6 cells (Figure 3C).

### Effect of cytokines on the invasion ability of B16/BL6 cells

**Figure 4:**
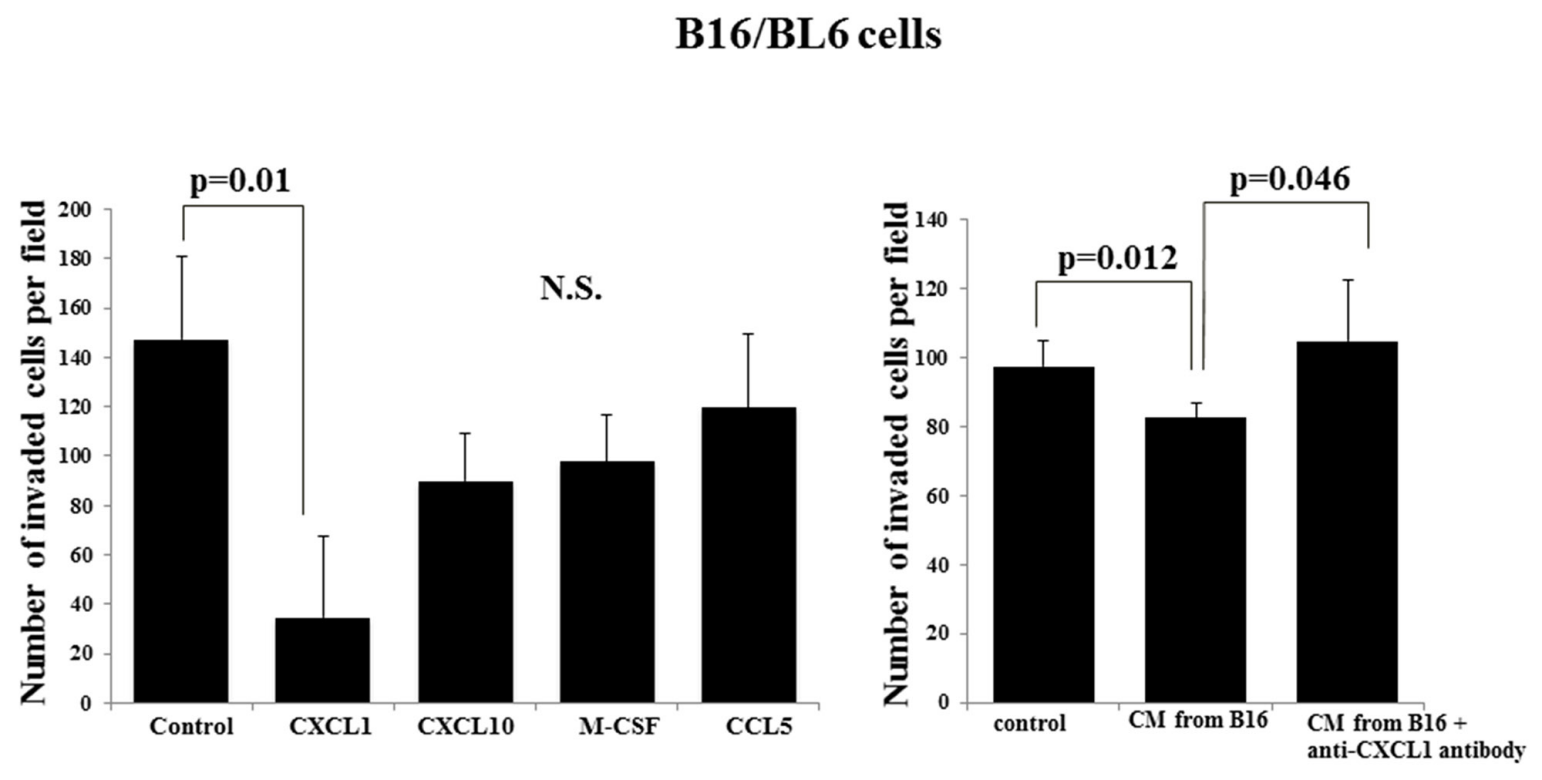
Effect of cytokines on the invasion ability of B16/BL6 cells CXCL1 significantly suppressed the invasion ability of B16/BL6 cells (p=0.01). CXCL10, M-CSF and CCL5 did not affect the invasion ability of B16/BL6 cells. CM from B16 significantly inhibited the invasion ability of B16/BL6 cells (p=0.012). The neutralizing anti-CXCL1 antibody significantly canceled the invasion-inhibitory effect of CM from B16 (p=0.046). The results are the mean of four independent experiments. Bars: SD.

CXCL1 significantly decreased the invasion ability of B16/BL6 cells (p=0.01), but CXCL10, M-CSF, and C-C motif chemokine 5 (CCL5) did not. The CM from B16 significantly decreased the invasion ability of B16/BL6 cells (p=0.012). The invasion-inhibitory activity of the CM from B16 was significantly suppressed by the neutralizing anti-CXCL1 antibody (p=0.046). In contrast, CXCL10, M-CSF, and CCL5 did not affect the invasion ability of B16/BL6 cells (Figure 4). CXCL1 did not affect the proliferation of the B16/BL6 cells (Supplementary Figure 1B).

### Effects of CXCL1 and CM from B16 on the expression levels of E-cadherin and N-cadherin in B16/BL6 cells

**Figure 5:**
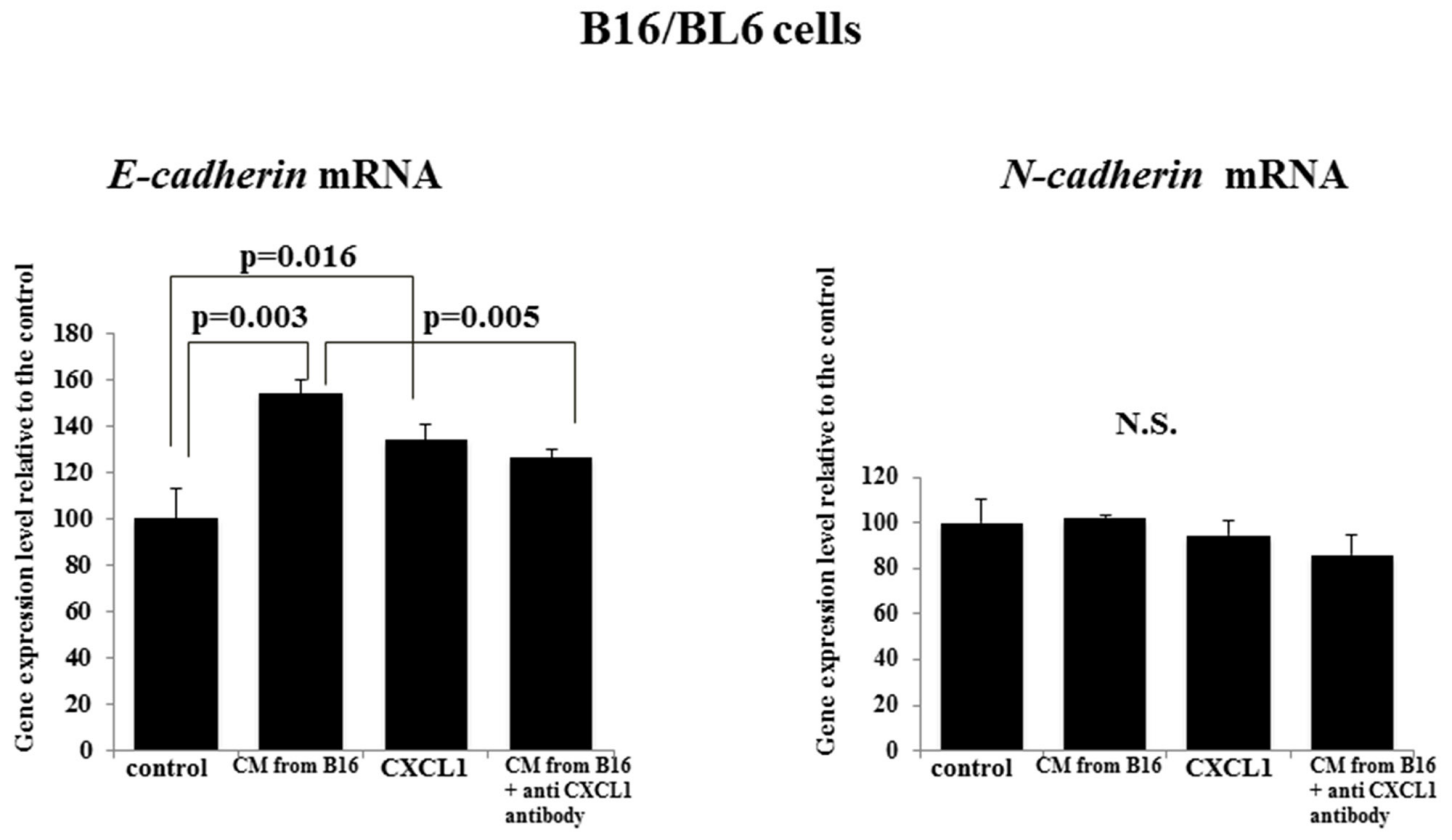
Effect of CXCL1 and that of the CM from B16 on *E-cadherin* and *N-cadherin* mRNA expression of B16/BL6 cells CM from B16 and CXCL1 both significantly increased *E-cadherin* mRNA compared to the control. Anti-CXCL1 neutralizing antibody decreased the *E-cadherin* mRNA level that had been increased by the B16 CM. CXCL1, CM from B16, and anti-CXCL1 neutralizing antibody each had no effect on the N-*cadherin* level. The results are the mean of three independent experiments. Bars: SD.

The *E-cadherin* mRNA level of the B16/BL6 cells was significantly increased by the CM from B16 (p=0.003) and by CXCL1 (p=0.016) compared with the control. Anti-CXCL1 neutralizing antibody significantly decreased the *E-cadherin* level that had been increased by the CM from B16 (p=0.005). In contrast, CXCL1 and CM from B16 did not affect the *N-cadherin* mRNA level of B16/BL6 cells (Figure 5).

### Effects of CXCL1 and CM from B16 on the MMP production by B16/BL6 cells

**Figure 6:**
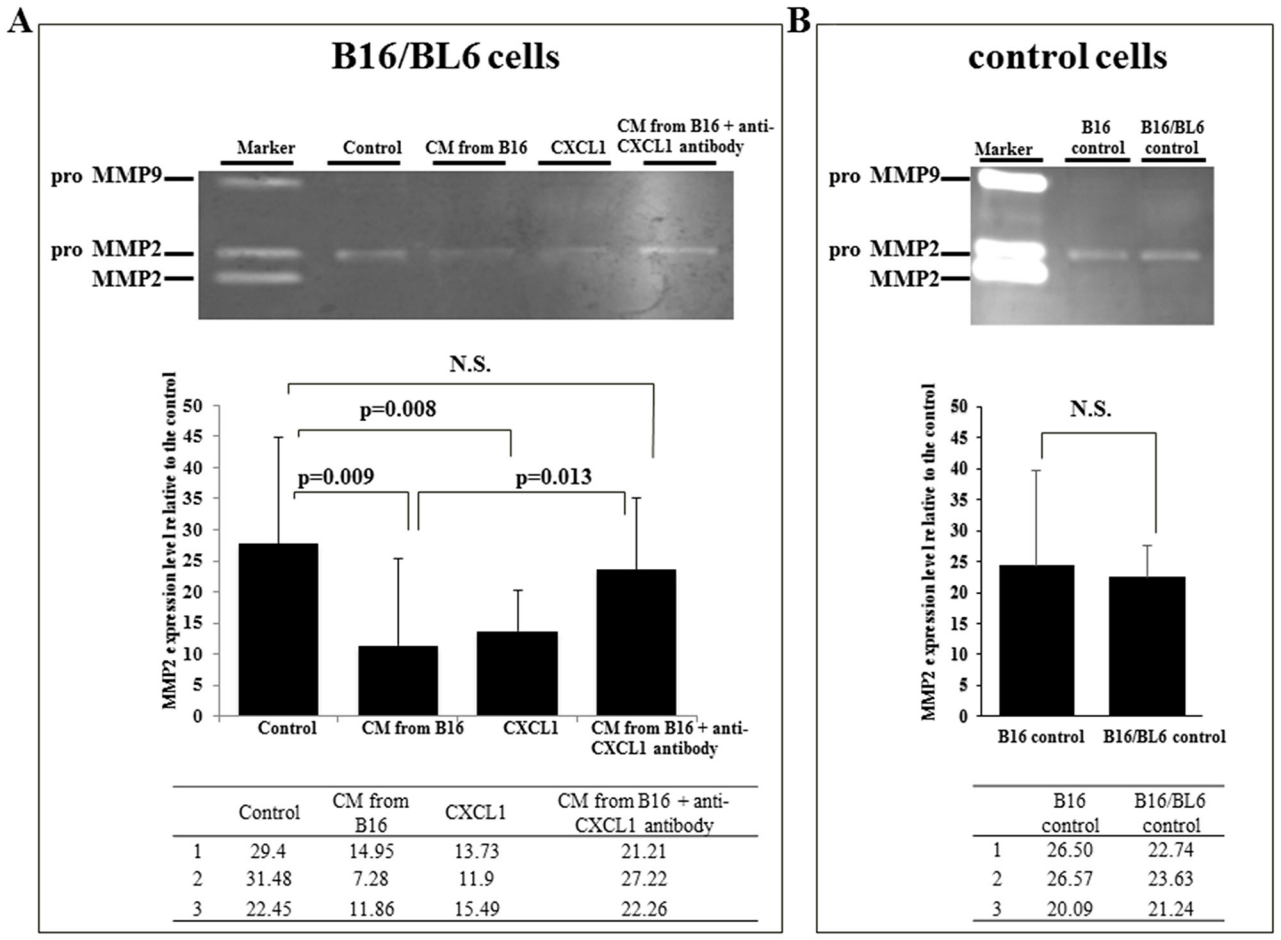
Effect of CXCL1 and CM from B16 on MMP2 activity of B16/BL6 cells **(A)** Representative picture of gelatin zymograph of B16/BL6 cells. Gelatin zymography showed that MMP2 activity of B16/BL6 cells was significantly decreased following the addition of CM from B16 (p=0.009) and CXCL1 (p=0.008). Anti-CXCL1 neutralizing antibody significantly increased the MMP2 level treated by CM from B16 (p=0.013). The results are the mean of three independent experiments. Bars: SD. **(B)** MMP2 activity of B16 cells and B16/BL6 cells. MMP2 activity was not significantly different between the control B16 cells and the control B16/BL6 cells.

The gelatin zymography showed that the CM from B16 and CXCL1 both significantly decreased the pro-MMP2 production by B16/BL6 cells (p=0.009, p=0.008, respectively). Anti-CXCL1 neutralizing antibody significantly increased the MMP2 production by B16/BL6 cells treated with CM from B16 (p=0.013) (Figure 6A). No significant difference in pro-MMP2 production was observed between the B16/BL6 CM and the B16 CM (Figure 6B).

## DISCUSSION

We examined the interaction between primary melanoma (B16) cells and the metastatic melanoma (B16/BL6) cells. Our results demonstrated that the primary melanoma cells produced the invasion-inhibitory factor which suppressed the invasion ability of the metastatic melanoma cells. Primary tumors were reported to limit metastatic formation; Kirstein *et al.* [6] and Kubo *et al.* [7] demonstrated the involvement of interleukin (IL)15 or platelets in the suppression of metastases using the highly metastatic melanoma cell line B16F10. Because our present data indicated that CM from primary B16 melanoma cells down-regulated the invasion ability of daughter metastatic melanoma cells B16/BL6, we next aimed to clarify the paracrine factor(s) from B16 cells that suppress the invasion ability of B16/BL6 cells.

The difference in production levels between a primary tumor and a metastatic tumor might be associated with the cell-cell interaction in the invasion process. In this study, the B16 melanoma cells produced different levels of CXCL1, CXCL10, M-CSF, and CCL5 compared to the B16/BL6 cells. Among these cytokines, only CXCL1 showed the invasion-inhibitory activity of B16/BL6 cells. It was reported that CXCL1 is increased in malignant melanoma cells and is involved in the metastasis of malignant melanoma [9, 10]. The production level of CXCL1 differs among human melanoma cell lines [11]. Our present findings suggest that CXCL1 from the primary tumor controlled the behavior of metastatic cancer cells in a paracrine manner.

CXCL1 and the CM from B16 increased the *E-cadherin* mRNA and decreased the MMP2 level of B16/BL6 cells. The up-regulation of E-cadherin expression was reported to inhibit the invasive ability of B16/BL6 cells [12] and B16F10 cells [13]. It was also reported that the suppression of MMP2 inhibited the invasion ability of B16F10 cells [14]. These findings suggested that CXCL1 in conditioned medium from B16 might inhibit the invasion ability of B16/BL6 cells by an up-regulation of E-cadherin and a down-regulation of MMP2. In contrast, the MMP2 level was not different between B16 cells and B16/BL6 cells, suggesting that the invasion potential might not be different between the B16 cells and B16/BL6 cells. CXCL1 from primary melanoma cells, B16, might inhibit the MMP2 activity of metastatic melanoma cells, B16F10. The molecular mechanisms clarified in the present study indicate that the CXCL1 signal might be a beneficial target for the development of a novel therapy against melanoma.

The conditioned medium from B16 cells affected the morphologic features of B16/BL6 cells, changing the cells’ shape from bipolar to multipolar. E-cadherin facilitates the morphology of tumor cells. The multipolar shape might be a feature of mesenchymal-epithelial transition. It has been reported that the morphological changes elongating with long and slim pseudopodia-like protrusions inhibit the migration and invasion abilities of melanoma cells, B16F10 cells [15]. The multipolar shape of B16/BL6 cells by CM from B16 might be associated with the pseudopodia-like protrusions.

We used a parent melanoma cell line and a daughter subline with high metastatic potential in this study, because no other pair of parent/daughter cell lines is available in melanoma. In the future, the additional establishment of pairs of parent/daughter melanoma cell lines might be necessary to confirm the significance of CXCL1 in the cell-cell interaction of invasion.

In conclusion, our study indicates that primary melanoma cells might down-regulate the invasion activity of metastatic melanoma cells through CXCL1 signaling.

## MATERIALS AND METHODS

### Cell lines

Two malignant melanoma cell lines, B16 and B16/BL6, were provided by the Riken Cell Bank (Ibaragi, Japan). B16/BL6 is a subline of B16 melanoma, with highly metastatic potential to the lungs of syngeneic C57BLi6N mice [16, 17]. The culture medium consisted of Dulbecco’s modified Eagle’s medium (DMEM) (Wako, Osaka, Japan) with the addition of 10% fetal bovine serum (FBS) (Nichirei Biosciences, Tokyo), 100 IU/mL penicillin (Wako), 100 mg/mL streptomycin (Wako), and 0.5 mM sodium pyruvate (Wako). Cells were cultured at 37°C in 5% CO_2_.

### Preparation of conditioned medium (CM)

We prepared the conditioned medium (CM) from B16 cells and from B16/BL6 cells as follows. To obtain CM, semi-confluent B16 and B16/BL6 cells in 100-mm plastic dishes were washed with phosphate-buffered saline (PBS; Wako) and incubated for an additional 3 days in 5 ml of serum-free DMEM. The supernatant was collected and stored as CM at -20°C until use. All experiments were performed in medium containing 1.5% FCS. As a control, DMEM was used instead of CM.

### MTT assay

The proliferation of B16 cells and that of B16/BL6 cells were determined by a 3- (4,5-dimethylthiazol-2-yl)-2,5-diphenyltetrazolium bromide (MTT) assay. Cells were seeded at 1×10^3^ per well in 96-well plates with the CM and incubated for 72 hr. Subsequently, 100 μl of culture medium and 20 μl of MTT solution (Promega, Tokyo) were added to each well, and the absorbance at 570 nm was analyzed using a microplate reader (Bio-Rad 550; Bio-Rad, Tokyo).

### Compounds

We used CXCL1, CXCL10, macrophage colony-stimulating factor (M-CSF), CCL5 anti-CXCL1-neutralizing antibody, all of which were purchased from Wako.

### Invasion assay

The *in vitro* invasiveness of cells was measured by a two-chamber Matrigel invasion assay. We used a chamber (Falcon) with an 8-μm-pore membrane filter coated with 50 μg of Matrigel (upper chamber) in a 24-well culture plate (lower chamber). B16/BL6 cells (2.5×10^4^ cells/500 μl /chamber) were seeded in the upper chamber, and 500 μl CM from B16 at a final concentration of 50 % CM with or without anti-CXCL1-neutralizing antibody (500 ng/ml), CXCL1 (10 ng/ml), CXCL10 (10 ng/ml), M-CSF (10 ng/ml), or CCL5 (10 ng/ml) was added to the low chamber. After incubation for 24 hr, B16/BL6 cells that invaded through the membrane were stained by Diff-Quick (Sysmex, Kobe, Japan) and were manually counted under a microscope at ×200 magnification. Six randomly chosen fields were counted, and the mean of the six fields was calculated as the sample value.

### Cell morphology

B16/BL6 cells were seeded and incubated for 7 days in the presence of 50% CM from B16 with 1.5% FBS. The morphology of the B16/BL6 cells was then observed under a light microscope.

### Cytokine array

The cytokine production from B16 cells and that from B16/BL6 cells were examined using the Mouse Cytokine Array Panel A (R&D Systems, Minneapolis, MN) according to the manufacturer’s protocol. CM from each cell line was mixed with a cocktail of biotinylated detection antibodies. After the streptavidin-horseradish peroxidase and chemiluminescent detection reagents were added, the chemiluminescence signal was evaluated.

### Reverse transcription-polymerase chain reaction (RT-PCR) analysis

A total of 1×10^5^ B16/BL6 cells were cultured under the respective conditions for 24 hr. After the extraction of mRNA from B16/BL6 cells, cDNAs were synthesized with ReverTra Ace qPCR RT Master Mix (Toyobo, Osaka, Japan). Relevant cDNA was amplified by PCR with Taq DNA polymerase (Nippon Gene, Tokyo) in a thermal cycler, with 40 cycles used for each of the three repeated steps. Primers for *E-cadherin*: Forward 5′-CGAGAGTCAGCCTTTAACGAAATG-3′ Reverse 5′-GGTCTTCCATTACGGAGAGATCC-3′, *N-cadherin;* Forward 5′-CGAGAGTCAGCCTTTAACGAAATG-3′ Reverse 5′-GGTCTTCCATTACGGAGAGATCC-3′, customized from Sigma-Aldrich (St. Louis, MO). *E-cadherin* and *N-cadherin* were electrophoresed with agarose gels, and the expression levels of E-cadherin and N-cadherin were measured. All quantitative RT-PCRs were done in triplicate.

### Gelatin zymography

A total of 1×10^5^ B16/BL6 cells/well were cultured under the respective conditions for 5 days. The protein was extracted from the cells. A gelatin-zymography kit (Cosmo Bio, Tokyo) was used to measure matrix metalloproteinases 2 (MMP2) and MMP9. A total of 10 μg of protein was added with sample buffer without reducing agent and then subjected to sodium dodecyl sulfate-polyacrylamide gel electrophoresis (SDS-PAGE) with a gel containing 0.1% gelatin. After electrophoresis, the gel was incubated in substrate buffer and then stained with Coomassie blue. The intensity of the bands was determined on a computerized densitometer LAS 3000 densitometer (GE Healthcare Life Sciences, Piscataway, NJ). Gelatin zymography was carried out three times.

### Statistical analysis

Data are expressed as mean ± SD, and differences in data were analyzed using the unpaired Student’s *t*-test. P-values <0.05 were accepted as significant.

## SUPPLEMENTARY MATERIALS FIGURE


