# Supplementary material for: C-X-C Motif Ligand 1 (CXCL1) from melanoma cells down-regulates the invasion of their metastatic melanoma cells
Source: Oncotarget. 2018 Jul 24;9(57):31090–7. doi: 10.18632/oncotarget.25783 (PMC6089562; doi:10.18632/oncotarget.25783)
Supplement: Supplementary file 1 [file oncotarget-09-31090-s001.pdf]

C-X-C Motif Ligand 1 (CXCL1) from melanoma cells down-regulates the invasion of their metastatic melanoma cells

SUPPLEMENTARY MATERIALS

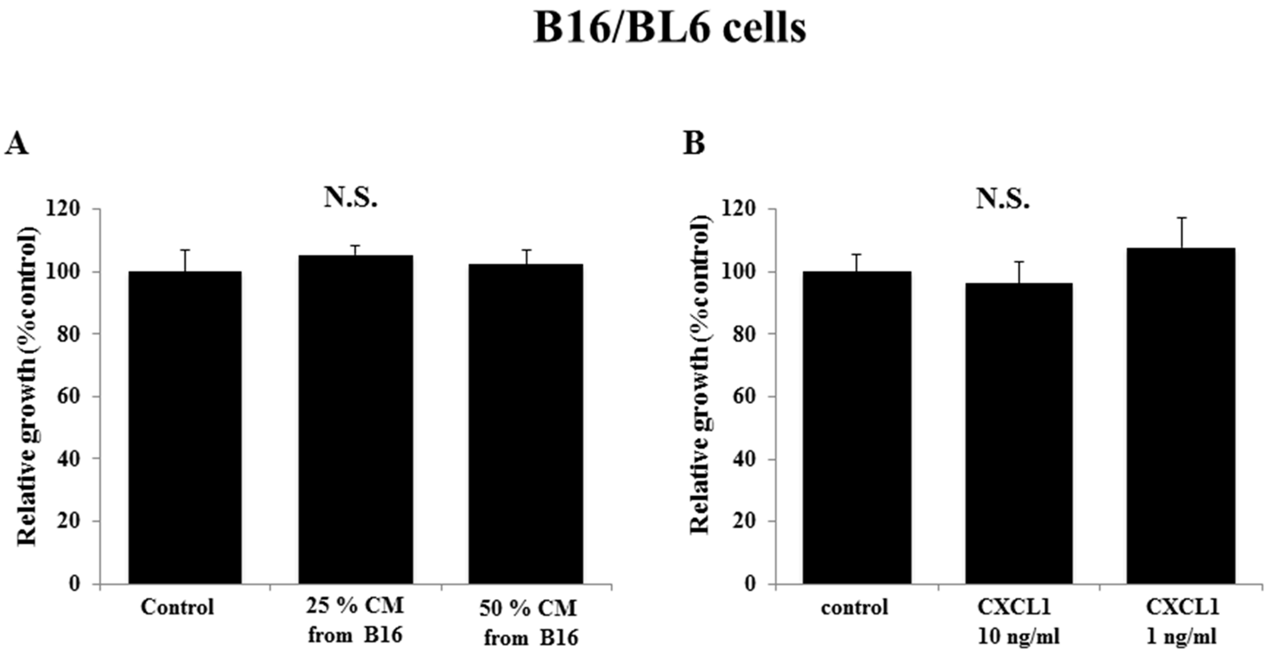

Supplementary Figure 1: Effect of CM from B16 and CXCL1 on the proliferation of B16/BL6 cells. **(A)** CM from B16 did not affect the proliferation of B16/BL6 cells. **(B)** CXCL1 did not affect the proliferation of B16/BL6 cells. The results are the mean of three independent experiments. Bars: SD.
